# Effects of follicular output rate on cumulative clinical pregnancy rate and cumulative live birth rate in PCOS patients with different characteristics

**DOI:** 10.3389/fendo.2022.1079502

**Published:** 2022-12-19

**Authors:** Rulan Jiang, Mingya Cao, Haomeng Hao, Rui Jia, Peipei Chen, Yuanyuan Liu, Zhiming Zhao

**Affiliations:** ^1^ Department of Reproductive Medicine, The Second Hospital of Hebei Medical University, Shijiazhuang, China; ^2^ Shenzhen Key Laboratory of Reproductive Immunology for Peri-Implantation, Shenzhen Zhongshan Institute for Reproduction and Genetics, Shenzhen Zhongshan Urology Hospital, Shenzhen, China; ^3^ Department of Gynecology and Obstetrics, Handan First Hospital, Handan, China

**Keywords:** follicular output rate, cumulative clinical pregnancy rate, cumulative live birth rate, polycystic ovarian syndrome, PCOS characteristics, *in vitro* fertilization-embryo transfer

## Abstract

**Objective:**

We aim to explore the effects of follicular output rate (FORT) on cumulative clinical pregnancy rate (CCPR) and cumulative live birth rate (CLBR) in polycystic ovary syndrome (PCOS) patients with different characteristics undergoing *in vitro* fertilization (IVF) treatment.

**Methods:**

This retrospective study analyzed 454 patients with PCOS undergoing their first IVF cycle at our center from January 2016 to December 2020. FORT was calculated as pre-ovulatory follicle count (PFC) × 100/antral follicle count (AFC). Multivariate regression analyses were conducted to explore the relationships between FORT and CCPR and CLBR. Curve fitting and threshold effect analyses were established to find nonlinear relationships. Effect modification in different subgroups were examined by stratification analyses.

**Results:**

Based on the FORT values, individuals were classified into the following three groups: low-FORT group, middle-FORT group and high-FORT group. Multivariate regression analyses revealed that FORT was an independent factor affecting the CCPR and CLBR significantly (OR = 1.015, 95% CI: 1.001, 1.030 and OR = 1.010, 95% CI:1.001, 1.020). Curve fitting and threshold effect analyses showed that the CCPR and CLBR had a positive correlation with FORT when the FORT was less than 70% (OR = 1.039, 95% CI: 1.013, 1.065 and OR = 1.024, 95% CI: 1.004, 1.044). Stratification analyses showed that the CLBR increased by 1.3% with each additional unit of FORT for patients with hyperandrogenic manifestations (OR = 1.013, 95% CI: 1.001, 1.025). Compared with the low-FORT group, in the high-FORT group, CCPR increased 1.251 times for patients with polycystic ovarian morphology, while CCPR and CLBR increased 1.891 times and 0.99 times for those with ovulation disorder, respectively (OR = 2.251, 95% CI: 1.008, 5.028 and OR = 2.891, 95% CI: 1.332, 6.323 and OR = 1.990, 95% CI: 1.133, 3.494).

**Conclusion:**

In patients with PCOS, cumulative IVF outcomes have a positive correlation with FORT when the FORT is less than 70%. For PCOS patients with polycystic ovarian morphology, ovulation disorder or hyperandrogenic manifestations, a high FORT could be conductive to achieving better pregnancy outcomes.

## Introduction

Polycystic ovary syndrome (PCOS) typically manifests with hyperandrogenism, oligo-anovulation and polycystic ovarian morphology. Studies have shown that PCOS is the main cause of anovulatory infertility, affecting about 8-13% of childbearing age women (1–3). For PCOS patients, *in vitro* fertilization (IVF) treatment is an assisted reproductive option, where controlled ovarian stimulation (COS) is the main step. However, COS in these patients frequently leads to large quantities of poor-quality oocytes and increased incidence of ovarian hyperstimulation syndrome (OHSS).

Anti-Mullerian hormone (AMH), antral follicle count (AFC), and basal follicle stimulating hormone (FSH) levels have been used to adjust ovarian stimulation (OS), minimize risks and optimize assisted reproductive technology (ART) outcomes, but all of them have certain limitations (4–7). They do not reflect the dynamic nature of follicular growth in response to exogenous gonadotrophins (Gn) and their ability to predict clinical outcomes after ART is limited (7, 8).

In 2011, Genro et al. (9) proposed the concept of the follicular output rate (FORT), the ratio of pre-ovulatory follicle count (PFC) on the trigger day to the AFC, to quantify the follicular development potential. Gallot et al. (10) further explored the correlation between pregnancy rate and FORT in patients who underwent IVF treatment with regular menstrual cycles. They found that better pregnancy outcomes were related to high FORT values. In addition, Hassan et al. (11) studied women with unexplained infertility and found that FORT had an independent effect on clinical pregnancy rate.

Several studies have suggested that FORT may reflect clinical outcomes after ART in PCOS patients, but consensus has not been reached (12–14). In an early study of 140 patients with PCOS, the fertilization rate and high quality embryo rate were highest in the middle-FORT group, although the FORT groups did not differ significantly in the clinical pregnancy rate (12). However, Tan et al. (13) found that the clinical pregnancy rate and high quality embryo rate increased with FORT in PCOS patients. A recent study of PCOS patients performed by Yang et al. (14) showed that the cumulative live birth rate (CLBR) was highest in the high-FORT group but lowest in the middle-FORT group.

Although Yang et al. (14) investigated the association of FORT with cumulative ART outcomes, the impact of clinical characteristics of PCOS was not taken into account in this study. A previous research showed that PCOS patients with different phenotypic characteristics reflected varied ovarian responses to COS (15). Furthermore, AFC and CLBR were significantly different in PCOS patients with different phenotypic features (15–17). To date, conclusive and definite data about the role of clinical characteristics of PCOS on the relationship between FORT and cumulative ART outcomes are still lacking.

In this study, we investigated the relationship between FORT and cumulative ART outcomes in PCOS patients with different characteristics after one IVF cycle including all fresh and subsequent frozen-thaw embryos, in order to guide COS medication and help PCOS patients get better reproductive outcomes.

## Materials and methods

### Subjects

This was a retrospective study of 454 PCOS patients undergoing the first IVF cycle from January 2016 to December 2020 in the Second Hospital of Hebei Medical University. The diagnosis of PCOS was assessed by the Rotterdam criteria (18), which requires at least two of the following: (1) oligoanovulatory ovarian dysfunction (OAD); (2) biochemical or clinical evidence of hyperandrogenism (HA); (3) polycystic ovarian morphology (PCOM). Exclusion criteria included endocrine abnormalities (such as abnormal thyroid function or Cushing’s syndrome), uterine cavity abnormalities, endometrial diseases, histories of ovarian surgery, chromosomal abnormalities, oocyte freezing, female age >38 years. Also, women who did not get a live birth and did not run out of all embryos were excluded. This study was approved by our hospital ethical committee.

### Treatment protocol

Patients adopted the gonadotropin-releasing hormone antagonist (GnRH-ant) protocol, gonadotrophin-releasing hormone agonist (GnRH-a) long protocol or GnRH-a prolonged protocol. In the GnRH-ant protocol, ovarian stimulation was started from the 2nd or 3rd day of the menstrual cycle with Gn (Recombinant Human Follitropin Alfa, MerckSerono, Italy, 75 IU) until one follicle reached 14 mm or more than six follicles reached 11-13 mm in diameter or serum E2 reached 400pg/ml, then GnRH-ant (Cetrorelix, MerckSerono, Switzerland, 0.25 mg) was administered daily. In the GnRH-a long protocol, triptorelin (Decapeptyl, Ferring, Germany, 1 ml; 0.1 mg) was used for pituitary down regulation, 0.1 mg once daily, from the middle luteal phase of the previous menstrual cycle. When the down regulation was confirmed, Gn was administered until triggering for oocyte maturation. In the GnRH-a prolonged protocol, GnRH-a (Decapeptyl, Ferring, Germany, 3.75mg) was injected in early follicular phase, and Gn was initiated 28-30 days later along with confirmation of pituitary down regulation.

For all the COS protocols, blood tests and ultrasound were used to monitor hormone levels and follicle growth. When the diameter of the leading follicle reached 18 mm or more than two follicles reached 17 mm, human chorionic gonadotropin (hCG) or GnRH agonists was used to trigger the oocyte final maturation referred to patients’ hyperstimulation risk. Oocytes were collected under ultrasound scan 36–37h after triggering.

Collected oocytes from each woman were inseminated through conventional IVF and fertilization assessment was carried out 17h after insemination. Based on the Istanbul Consensus (19) and Vienna Consensus (20), the embryos were classified into grades I-IV referred to their morphology, cell number and the percentage of fragmentation at 72h after fertilization. Grade I embryos on day 3 of culture were taken as high quality embryos. Grade I-II embryos on day 3 were considered available embryos which could be transferred or frozen. The remaining cleavage embryos were cultured to blastocysts and those with a Gardner score above 3CC on day 5 or 6 were considered suitable for vitrification.

For fresh embryo transfer, the embryo transfer was carried out on the 3rd or 5th day following oocyte retrieval. For frozen embryo transfer, the patients started taking 2-3 mg of oral estradiol twice daily from the third day of the menstrual cycle for endometrial preparation. Vaginal progesterone was administered for corpus luteum support when the thickness of the endometrium reached 8 mm under ultrasound scan. Frozen embryo transfer was scheduled on the 4th or 6th day of corpus luteum support.

After embryo transfer, progesterone was given for corpus luteum support. The β-hCG level in peripheral blood was measured 2 weeks after embryo transfer. The transvaginal ultrasound examination of the gestational sac and heart beat was carried out 28-30 days following embryo transfer. Clinical pregnancy was defined as one or more intrauterine gestational sacs with heartbeat visualized under ultrasound scan.

### Observation indicators

The AFC was recorded with a diameter of 3–10 mm at baseline. On the trigger day, the PFC was recorded with a diameter of 14–22 mm. FORT was calculated as PFC×100/AFC. The main outcomes of our study were cumulative clinical pregnancy rate (CCPR) and CLBR. CCPR was calculated as the number of clinical pregnancy cycles/number of first oocyte retrieval cycles. CLBR was calculated as the number of live birth cycles/number of first oocyte retrieval cycles.

### Statistical analysis

We performed all statistical analyses with SPSS26.0 software and EmpowerStats (X&Y solutions, Inc., Boston, MA). Continuous variables were presented as mean ± SD or median (Q1-Q3). Categorical variables were presented as percentages.

If the variables were in normal distribution, variance analysis method and two-independent sample test were conducted for group comparisons. If the variables followed non-normal distribution, non-parametric Mann-Whitney U tests were applied to compare continuous variables. Fisher’s exact test or Chi-square test was performed when comparing categorical variables. Univariate analyses were conducted with logistic regression models to detect the possible variables which may affect cumulative ART outcomes. Multivariate logistic regression analyses were carried out to estimate the associations between FORT and CCPR and CLBR. Curve fitting and threshold effect analyses were established to find nonlinear relationships. The relationships between FORT and cumulative ART outcomes in different subgroups were examined by stratification analyses. A *p*-value <0.05 was considered significant statistically.

## Results

### Baseline status

**Figure 1:**
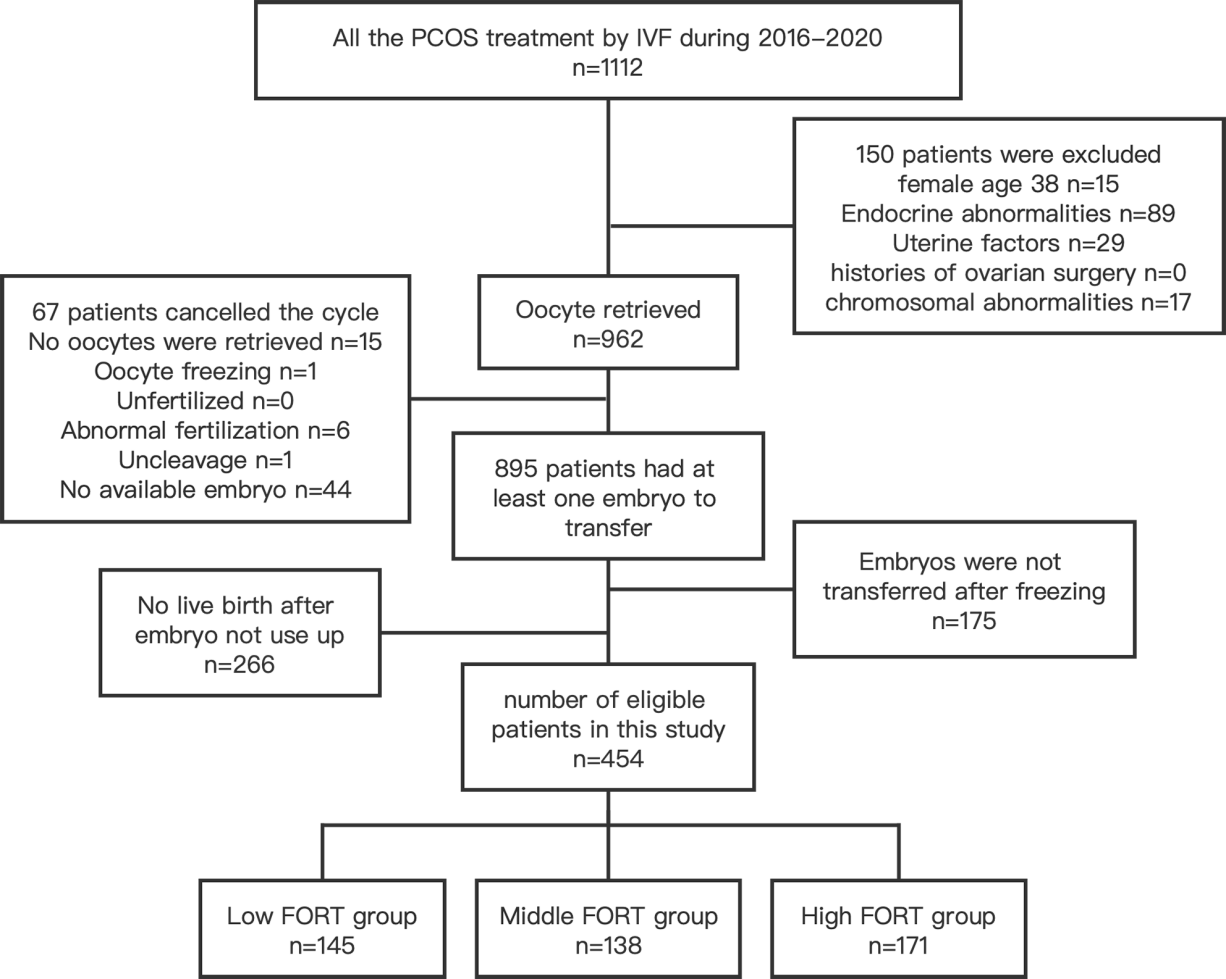
Flow chart for selection of patients from January 2016 to December 2020.

There were 454 PCOS patients included in our study after exclusions (**Figure 1**). Based on the FORT values, individuals were classified into the following three groups: low-FORT group (n = 145) with FORT values below the 33rd percentile (FORT <0.54), middle-FORT group (n = 138) with FORT values between the 33rd and 67th percentiles (FORT 0.54~0.66), and high-FORT group (n = 171) with FORT values above the 67th percentile (FORT >0.66). **Table 1** showed the patients’ baseline characteristics. The age, body mass index (BMI), years of infertility, basal estradiol (E_2_), basal FSH, starting dose of Gn, dose of Gn and duration of Gn were similar among the three groups. The AFC in the high-FORT group was lower than that in the middle-FORT and low-FORT group (*p* < 0.05). The AMH in the high-FORT group was higher than that in the low-FORT group (*p* < 0.05). The basal testosterone (bT), PFC were significantly higher in the high-FORT group than in the middle-FORT group and low-FORT group (*p* < 0.05). Patients with the high basal testosterone level accounted for a large proportion in the high-FORT group (62.573%). **Table 2** showed the laboratory indicators and clinical outcomes of study participants. The number of retrieved oocytes, number of 2PN zygotes and number of cleavage embryos in the high-FORT group were significantly higher than that in the middle-FORT and low-FORT group (*p* < 0.05). The fertilization rate, available embryo rate, number of available embryos and number of high-quality embryos were significantly higher in the high-FORT group than in the low-FORT group (*p* < 0.05). The CCPR significantly increased with FORT (*p* < 0.05). The CLBR increased with increasing FORT although the *p*-value was not significant.

**Table 1:** Patient’s baseline characteristics.

FORT	Low (<0.54, n=145)	Middle (0.54~0.66, n=138)	High (>0.66, n=171)	*F/χ^2^*	*p-*value
Age (years)	28.269 (2.935)	28.725 (3.311)	28.661 (3.310)	0.869	0.420
BMI (kg/m2)	25.650 (3.258)	25.416 (3.595)	24.777 (3.498)	2.740	0.066
Years of infertility (years)	3.000 (2.000-5.000)	3.000 (2.000-5.000)	3.000 (2.000-4.000)	2.941	0.054
bFSH (mIU/mL)	6.905 (1.869)	6.542 (1.708)	6.489 (1.773)	2.415	0.091
bE2 (pg/mL)	41.000(28.000-59.000)	42.000 (32.000-60.000)	41.000 (30.000-57.000)	0.260	0.771
bP (ng/mL)	0.650 (0.390-0.970)	0.665 (0.472-1.015)	0.650 (0.390-1.010)	0.495	0.61
bLH (mIU/mL	8.060 (4.740-12.560)	8.185 (4.855-11.985)	8.280 (4.145-13.270)	0.462	0.631
bT (ng/mL)	0.650 (0.490-0.820)*	0.660 (0.490-0.835)*	0.750 (0.595-0.880)	8.181	<0.001
AMH (ng/mL)	5.820 (4.170-8.810)*	6.405 (4.720-9.158)	7.050 (4.960-11.260)	5.276	0.005
No. of AFC	23.579 (4.445)*	23.203 (2.365)*	21.064 (4.865)	17.406	<0.001
Starting dose of Gn (IU	178.017 (55.282)	171.830 (45.811)	176.681 (47.704)	0.609	0.545
Dose of Gn (IU	2250.000 (1650.000-2925.000)	2193.750 (1650.000-2765.625)	2000.000 (1500.000-2475.000)	3.023	0.050
Duration of Gn (IU)	11.262 (2.789)	11.797 (4.215)	11.029 (2.753)	2.152	0.163
No. of PFC	10.159 (2.394)*#	13.529 (1.567)*	18.789 (7.028)	141.728	<0.001
FORT	45.830 (37.500-50.000)*#	58.330 (54.170-62.500)*	79.170(70.830-100.000)	274.479	<0.001
Type of infertility				0.824	0.662
Primary	93 (64.138%)	83 (60.145%)	111 (64.912%)		
Secondary	52 (35.862%)	55 (39.855%)	60 (35.088%)		
Type of PCOS				28.464	<0.001
A	44 (30.345%)	39 (28.261%)	59 (34.503%)		
B	10 (6.897%)	7 (5.072%)	33 (19.298%)		
C	8 (5.517%)	11 (7.971%)	15 (8.772%)		
D	83 (57.241%)	81 (58.696%)	64 (37.427%)		
PCOM				19.454	<0.001
no	10 (6.897%)	7 (5.072%)	33 (19.298%)		
yes	135 (93.103%)	131 (94.928%)	138 (80.702%)		
OAD				1.266	0.531
no	8 (5.517%)	11 (7.971%)	15 (8.772%)		
yes	137 (94.483%)	127 (92.029%)	156 (91.228%)		
HA				17.361	<0.001
no	83 (57.241%)	80 (57.971%)	64 (37.427%)		
yes	62 (42.759%)	58 (42.029%)	107 (62.573%)		
Treatment plan				5.244	0.263
GnRH-ant protocol	33 (22.759%)	19 (13.768%)	35 (20.468%)		
GnRH-a long protocol	35 (24.138%)	35 (25.362%)	34 (19.883%)		
GnRH-a prolonged protocol	77 (53.103%)	84 (60.870%)	102 (59.649%)		
Cycle outcome				34.161	<0.001
Fresh embryo transfer	97(66.897%)	67(48.551%)	58(33.918%)		
Frozen embryo transfer	48(33.103%)	71(51.449%)	113(66.082%)		
Number of transferred cycles				4.507	0.342
1	136 (93.793%)	128 (92.754%)	152 (88.889%)		
2	9 (6.207%)	10 (7.246%)	19 (11.111%)		

#P<0.05 compared with middle group.

*P<0.05 compared with high group.

Categorical variables are presented as number (%). Continuous variables are presented as mean (SD) or median (interquartile range). BMI, body mass index; bFSH, basal follicle-stimulating hormone; bE2, baseline estradiol; bP, baseline progesterone; bT, baseline testosterone; bLH, baseline luteinizing hormone; AMH, anti-Mullerian hormone; Gn, Gonadotropin; PCOM, polycystic ovarian morphology; OAD, oligoanovulatory ovarian dysfunction; HA, hyperandrogenism.

**Table 2:** Patient’s laboratory indicators and clinical outcomes.

FORT	Low (<0.54, n=145)	Middle (0.54~0.66, n=138)	High (>0.66, n=171)	*F*/χ^2^	*p-*value
No. of oocyte	11.000 (8.000-17.000)*#	18.000 (13.000-22.750)*	19.000 (15.000-27.000)	35.612	<0.001
No. of 2PN	7.000 (4.000-10.000)*#	11.000 (7.000-15.000)*	12.000 (8.000-17.000)	32.156	<0.001
No. of cleavage embryo	9.000 (6.000-14.000)*#	15.000 (11.000-18.750)*	16.000 (12.000-23.000)	34.336	<0.001
No. of available embryo	3.000 (2.000-4.000)*#	4.500 (3.000-6.000)	5.000 (3.000-7.000)	14.353	<0.001
No. of high quality embryo	1.000 (0.000-2.000)*	1.000 (0.000-3.000)	1.000 (0.000-4.000)	4.845	0.008
Total number of transferred embryo	2.021 (0.520)	2.058 (0.509)	2.099 (0.620)	0.789	0.391
Fertilization rate	0.700 (0.213)*	0.733 (0.182)	0.762 (0.178)	4.027	0.018
2PN Fertilization rate	0.600 (0.440-0.730)	0.620 (0.500-0.750)	0.640 (0.500-0.775)	2.434	0.089
Cleavage rate	0.975 (0.124)	0.961 (0.152)	0.989 (0.032)	2.494	0.084
Available embryo rate	0.300 (0.220-0.400)*	0.270 (0.180-0.350)	0.250 (0.170-0.350)	5.462	0.005
High quality embryo rate	0.080 (0.000-0.200)	0.080 (0.000-0.190)	0.070 (0.000-0.180)	0.004	0.996
Cumulative clinical pregnancy	120 (82.759%)	124 (89.855%)	157 (91.813%)	6.688	0.035
Cumulative live birth	101 (69.655%)	101 (73.188%)	138 (80.702%)	5.397	0.067

#P<0.05 compared with middle group.

*P<0.05 compared with high group.

Categorical variables are presented as number (%). Continuous variables are presented as mean (SD) or median (interquartile range).

### Follicular output rate is an independent factor for IVF outcome

The consequences of the univariate analyses were given in **Supplementary Table 1**. The multivariate logistic regression analysis adjusted for the following confounders: age, BMI, years of infertility, AMH, treatment plan, type of PCOS. In the adjusted model, we found that the FORT was an independent factor significantly affecting the CCPR and CLBR (OR = 1.015, 95% CI: 1.001, 1.030 and OR = 1.010, 95% CI:1.001, 1.020) (**Tables 3**, **4**). The CCPR and CLBR increased by 1.5% and 1.0%, respectively, with each additional unit of FORT. The CCPR increased 2.017 times and the CLBR increased 1.188 times in the high-FORT group compared with the low-FORT group (OR = 3.017, 95% CI: 1.433, 6.355 and OR = 2.188, 95% CI: 1.256, 3.813).

**Table 3:** Follicular output rate and cumulative clinical pregnancy rate.

	Non-adjusted		Adjusted	
	OR (95%CI)	*p-*value	OR (95%CI)	*p*-value
FORT				
Low	1		1	
Middle	1.845(0.916, 3.719)	0.08663	2.008 (0.975, 4.138)	0.05871
High	2.336(1.165, 4.686)	0.01688	3.017 (1.433, 6.355)	0.00366
FORT	1.011(0.998, 1.024)	0.10493	1.015 (1.001, 1.030)	0.03496

Adjusted age, BMI, AMH, years of infertility, type of PCOS, treatment plan.

**Table 4:** Follicular output rate and cumulative live birth rate.

	Non-adjusted		Adjusted	
	OR (95%CI)	*p*-value	OR (95%CI)	*p*-value
FORT				
Low	1		1	
Middle	1.189(0.709, 1.994)	0.51119	1.275(0.749, 2.170)	0.37060
High	1.822(1.084, 3.062)	0.02356	2.188(1.256, 3.813)	0.00572
FORT	1.007(0.998, 1.016)	0.10747	1.010(1.001, 1.020)	0.03268

Adjusted age, BMI, AMH, years of infertility, type of PCOS, treatment plan.

**Figure 2:**
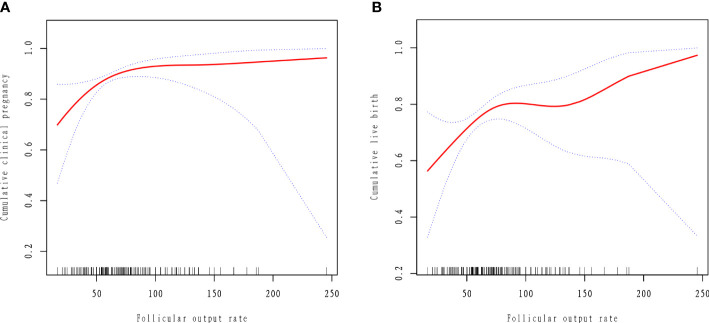
Curve fitting between follicular output rate and clinical outcomes. The adjusted smoothed plots between the follicular output rate with the cumulative clinical pregnancy rate and the cumulative live birth rate based on two-piece-wise regression model **(A, B)**. The nonlinear relationship between the follicular output rate and the cumulative clinical pregnancy rate and the cumulative live birth rate, respectively. Adjustment factors included age, BMI, AMH, years of infertility, type of PCOS, treatment plan. The solid line and dashed line represent the estimated values and their corresponding 95% confidence intervals.

The results of curve fitting revealed a curvilinear relationship between FORT and cumulative IVF outcomes, after adjustment for age, BMI, years of infertility, AMH, treatment plan and type of PCOS (**Figure 2**). Threshold effect analyses showed that the CCPR increased by 3.9% and the CLBR increased by 2.4% with each additional unit of FORT when the FORT was lower than 70% (OR = 1.039, 95% CI: 1.013, 1.065 and OR = 1.024, 95% CI: 1.004, 1.044) (**Table 5**). However, when the FORT was higher than 70%, the growth trend in the CCPR and CLBR with FORT was no longer significant (OR = 0.999, 95% CI: 0.983, 1.016 and OR = 1.003, 95% CI: 0.990, 1.015).

**Table 5:** The threshold effect analysis of the follicular output rate and clinical outcomes.

Cut points	N	OR	95%CI	*p*-value
The cumulative pregnancy rate				
< 70	316	1.039	(1.013, 1.065)	0.0029
>70	138	0.999	(0.983, 1.016)	0.9188
The cumulative live birth rate				
< 70	316	1.024	(1.004, 1.044)	0.0170
>70	138	1.003	(0.990, 1.015)	0.6813

Adjusted age, BMI, AMH, years of infertility, type of PCOS, treatment plan.

### Stratification analysis

Stratification analysis was performed separately based on age, BMI and clinical characteristics of PCOS. When the patients’ age was over 30, the CCPR increased by 3.3% and the CLBR increased by 1.6% with each additional unit of FORT (OR = 1.033, 95% CI: 1.005, 1.062 and OR = 1.016, 95% CI: 1.000, 1.033). When the patients were younger than 30, the CCPR and CLBR did not correlate significantly with FORT (**Table 6**).

**Table 6:** Stratification analysis of follicular output rate and cumulative clinical outcomes.

	N	The cumulative pregnancy rate		The cumulative live birth rate	
		OR (95%CI)	*p*-value	OR (95%CI)	*p*-value
Age					
<30	287	1.007 (0.990, 1.025)	0.4064	1.007 (0.995, 1.020)	0.2489
>=30	167	1.033 (1.005, 1.062)	0.0198	1.016 (1.000, 1.033)	0.0496
BMI					
<25	210	1.024 (1.002, 1.046)	0.0311	1.013 (1.001, 1.026)	0.0398
>=25	244	1.016 (0.996, 1.036)	0.1189	1.012 (0.997, 1.027)	0.1272

Adjustment factors included age, BMI, AMH, years of infertility, type of PCOS, treatment plan, if not stratified by its.

When BMI was lower than 25, the CCPR increased by 2.4% and the CLBR increased by 1.3% with each additional unit of FORT (OR = 1.024, 95% CI: 1.002, 1.046 and OR = 1.013, 95% CI: 1.001, 1.026). When BMI was 25 or larger than that, the CCPR and CLBR did not correlate significantly with FORT (**Table 6**).

Among patients with hyperandrogenic manifestations, the CLBR increased by 1.3% with each additional unit of FORT (OR = 1.013, 95% CI: 1.001, 1.025). The CCPR increased 1.541 times and the CLBR increased 1.451 times in the high-FORT group compared with the low-FORT group (OR = 2.541, 95% CI: 1.041, 6.202 and OR = 2.451, 95% CI: 1.169, 5.139). Among patients with polycystic ovarian morphology, the CCPR increased 1.251 times in the high-FORT group compared with the low-FORT group (OR = 2.251, 95% CI: 1.008, 5.028). Among patients with ovulation disorder, the CCPR increased 1.891 times and the CLBR increased 0.99 times in the high-FORT group compared with the low-FORT group (OR = 2.891, 95% CI: 1.332, 6.323 and OR = 1.990, 95% CI: 1.133, 3.494) (**Tables 7**, **8**).

**Table 7:** Stratification analysis of follicular output rate and cumulative clinical outcomes.

	N	The cumulative pregnancy rate		The cumulative live birth rate	
		OR (95%CI)	*p*-value	OR (95%CI)	*p*-value
PCOM					
No	50	1.007 (0.979, 1.036)	0.6339	1.005 (0.980, 1.029)	0.7173
Yes	404	1.014 (0.997, 1.031)	0.0983	1.010 (0.999, 1.021)	0.0821
OAD					
No	34	1.022 (0.974, 1.074)	0.3731	1.038 (0.986, 1.094)	0.1576
Yes	420	1.013 (0.998, 1.028)	0.0833	1.007 (0.998, 1.017)	0.1330
HA					
No	227	1.013 (0.986, 1.041)	0.3437	1.006 (0.990, 1.022)	0.4734
Yes	227	1.013 (0.997, 1.030)	0.1206	1.013 (1.001, 1.025)	0.0386

Adjusted age, BMI, AMH, years of infertility, treatment plan.

PCOM, polycystic ovarian morphology; OAD, oligoanovulatory ovarian dysfunction; HA, hyperandrogenism.

**Table 8:** Stratification analysis of follicular output rate and cumulative clinical outcomes.

	N	The cumulative pregnancy rate		The cumulative live birth rate	
		OR (95%CI)	*p*-value	OR (95%CI)	*p*-value
PCOM					
No					
Low FORT	10	1		1	
Middle FORT	7	10.270(0.270, 391.238)	0.2098	4.866 (0.265, 89.481)	0.2868
High FORT	33	11.392(0.692, 187.649)	0.0887	5.950 (0.735, 48.200)	0.0947
Yes					
Low FORT	135	1		1	
Middle FORT	131	1.816 (0.857, 3.852)	0.1196	1.166 (0.672, 2.021)	0.5857
High FORT	138	2.251 (1.008, 5.028)	0.0479	1.768 (0.978, 3.197)	0.0592
OAD					
No					
Low FORT	8	1		1	
Middle FORT	11	3.772 (0.198, 72.002)	0.3776	0.486 (0.041, 5.760)	0.5676
High FORT	15	1.848 (0.070, 48.735)	0.7130	7.929 (0.282,223.082)	0.2239
Yes					
Low FORT	137	1		1	
Middle FORT	127	1.862 (0.883, 3.925)	0.1025	1.345 (0.773, 2.342)	0.2946
High FORT	156	2.891 (1.322, 6.323)	0.0079	1.990 (1.133, 3.494)	0.0166
HA					
No					
Low FORT	83	1		1	
Middle FORT	80	1.588 (0.562, 4.484)	0.3824	1.136 (0.549, 2.353)	0.7309
High FORT	64	3.029 (0.730, 12.576)	0.1270	1.700 (0.715, 4.039)	0.2297
Yes					
Low FORT	62	1		1	
Middle FORT	58	2.161 (0.779, 5.996)	0.1388	1.271 (0.577, 2.801)	0.5523
High FORT	107	2.541 (1.041, 6.202)	0.0405	2.451 (1.169, 5.139)	0.0176

Adjusted age, BMI, AMH, years of infertility, treatment plan.

PCOM, polycystic ovarian morphology; OAD, oligoanovulatory ovarian dysfunction; HA, hyperandrogenism.

## Discussion

In this retrospective study of 454 PCOS patients, we found that FORT was an independent factor affecting the cumulative IVF outcomes. The CLBR and CCPR were positively correlated with FORT when the FORT was less than 70%.

FORT, as a simple and noninvasive tool in our clinical practice, could objectively reflect dynamic changes of follicular growth in response to exogenous Gn. Genro et al. found a negative association between FORT and AMH levels in peripheral blood, which might be explained by the hypothesis that AMH inhibited the sensitivity of antral follicles to Gn (9). Hassan et al. showed that no significant difference in the serum AMH levels was found among the FORT groups (11). We found that FORT was positively associated with AMH levels in peripheral blood. The difference between our results and other studies may be due to the disparities in the studied populations. We studied patients with PCOS, whereas Genro et al. and Hassan et al. studied patients with different infertility causes (9, 11). In the studies conducted on PCOS cases only, the serum AMH levels can be used as a marker of ovarian responsiveness and there was a positive association between AMH levels and assisted reproductive outcomes (21). In our study, basal testosterone levels in the high-FORT group were dramatically higher than that in the low-FORT and middle-FORT groups. This finding supports the previous studies which showed that basal testosterone level positively correlated with ovarian response and follicular count on trigger day (≥ 14 mm) (22, 23). The possible mechanism is that androgens could enhance FSH receptor expression in granulosa cells and are considered to promote follicular development by amplifying the effects of FSH (24). Additionally, androgens also augment the expression of insulin-like growth factor 1 (IGF-1) in the primate ovary, which is crucial for regulating follicular growth (22, 25).

This study found that the CCPR, cleavage embryos, 2PN zygotes, number of retrieved oocytes and PFC increased progressively from the low to high FORT groups. And the numbers of available embryos and high quality embryos were significantly lower in the low FORT group. These results are in agreement with the earlier reported results (10–12). In the high-FORT group, patients have better ovarian responsiveness to exogenous gonadotrophins, resulting in increased mature and retrieved oocytes, and consequently better clinical outcomes. In our study, the fertilization rate was significantly higher in the high FORT group. Our findings are in agreement with those of Hassan et al. (11), but they differ from that obtained in other studies (10, 12, 14), which did not show any difference in fertilization rate among the FORT groups. The difference between other studies and our findings may be attributed to the disparities in the number of cases investigated and study populations. We also found that the available embryo rate was higher in the low FORT group. This may be due to the relatively low number of retrieved oocytes in the low-FORT group. Despite these contradictions, the findings revealed that FORT can be used as a qualitative reflector of the follicular responsiveness to FSH, oocyte competence, and embryo quality.

The central finding of our study is the positive correlation between FORT and CCPR and CLBR in PCOS patients. The CCPR and CLBR in the high-FORT group were significantly higher than that in the low-FORT group. With the widespread use of embryo cryo-resuscitation technology, the CCPR and CLBR, defined as the pregnancy and live birth after using up all fresh and frozen embryos derived from one single COS cycle, appear to be more accurate and comprehensive measures to reflect the effectiveness and safety of IVF treatment (26–29). After reviewing the published literatures regarding PCOS and FORT, we found that there were few researches used CCPR and CLBR as clinical outcome indicators. Yang et al. (14) investigated the relationship between FORT and CLBR and showed that the CLBR was highest in the high-FORT group and lowest in the middle-FORT group. Differences between this study and our results may be due to the disparities in the study populations and the COS protocols. Also, in their study, there were significant differences in Gn dosage and stimulation days among three groups. This may affect the outcome because PCOS patients are usually high ovarian responders.

With a threshold effect model, our results showed that when the FORT was lower than 70%, the CCPR increased by 3.9% and the CLBR increased by 2.4% with each additional unit of FORT. When the FORT was greater than 70%, the CCPR and CLBR did not increase significantly even if the FORT increased. The positive association between the FORT and the cumulative ART outcomes in the first segment of the curve suggested the importance of the high FORT values for the success of IVF. Patients with high FORT values may have more oocytes and produce more euploid embryos that can be used for embryo transfer, thereby increasing the chance of pregnancy and live birth. However, when the FORT reached a certain value, there was no longer a significant beneficial relationship. This may be due to the fact that pregnancy outcomes are influenced by many other factors, such as obesity, environmental exposure (including smoking and alcohol), stress and antiphospholipid syndrome (30, 31). Therefore, for PCOS patients, we should also pay attention to the general conditions and lifestyle rather than simply increasing the FORT.

According to our results, patients with hyperandrogenemia (HA) can increase the CCPR and CLBR by increasing the FORT. HA is the core etiology and primary endocrine characteristic of PCOS. HA leads to premature granulosa cell luteinization and abnormal oocyte maturation by altering follicular fluid microenvironment and the feedback of ovarian hormones to hypothalamic-pituitary-ovarian (HPO) axis (32). In addition, due to the expression of androgen receptors in pancreas and hepatocytes, high testosterone levels could lead to hyperinsulinemia, which seriously impairs ovarian function resulting in premature arrest of follicular development and oligo-anovulation (33). Furthermore, high testosterone levels in PCOS patients also influence glucose metabolism of endometrium, which leads to local insulin resistance and subsequently endometrial lesion (34). Evidence show that the CLBR of patients with hyperandrogenemia is significantly lower than that of individuals without hyperandrogenemia (16). Therefore, improving FORT is a good choice for these poor-prognosis patients with HA to improve the CCPR and CLBR. Furthermore, we also found that patients with polycystic ovarian morphology or ovulation disorder had better cumulative IVF outcomes in the high-FORT group. These results together suggest that FORT, as a noninvasive and simple tool in our clinical practice, may contribute to improving the CCPR and CLBR for PCOS patients with typical clinical characteristics.

Our results showed that the CCPR and CLBR increased with FORT in PCOS patients over 30 years old, whereas the relationship was not statistically significant in patients under the age of 30. This indicated that the older the age, the more positive correlation between the FORT and cumulative ART outcomes. We know that oocyte quality gradually declines with women aging and the competence of women’s oocytes begins to deteriorate around their third decade (35). Multiple potential mechanisms may be responsible for this, such as meiotic spindle abnormalities, mitochondrial dysfunction and chronic exposure to oxidative stress, which usually lead to aneuploidy of the embryo and a higher incidence of adverse pregnancy outcomes (36–39). In general, younger women have better oocyte quality, which may somewhat attenuate the impact of FORT on pregnancy outcomes. Therefore, for the older age group of PCOS patients, due to the decline of oocyte quality, higher FORT values and more oocytes retrieved are needed to achieve better cumulative ART outcomes.

In this study, we found that the FORT was significantly related with the CCPR and CLBR in PCOS patients without overweight and obesity. By boosting FORT, we can increase the CCPR and CLBR in these individuals. Obesity and overweight are known risk factors for cumulative ART outcomes (40), and PCOS patients are more likely to be obese and overweight, which contributes to diminished response to ART, adverse pregnancy outcomes and higher incidence of other complications (41, 42). Obesity could impair endometrial function through inflammation, oxidative stress or other ways, which could cause decidual formation abnormalities and embryo implantation failure (43). Additionally, obesity affects oocyte function by inducing abnormal chromosome pairing and altering follicles’ liquid microenvironment (32). Therefore, weight control and alleviating metabolism disorders are more beneficial to the prognosis of overweight and obese patients than increasing the FORT.

The main strengths of our study rest on the following aspects. First, it was the first study to investigate the role of clinical characteristics of PCOS on the relationship between FORT and cumulative IVF outcomes. Second, the present study first uncovered a curvilinear relationship between FORT and CCPR and CLBR, and this relationship might be useful in establishing an optimal treatment strategy for PCOS patients to obtain better reproductive outcomes. Additionally, our research used CCPR and CLBR as main outcome measures, which is an important advantage over other metrics.

Despite the strengths, this study has some limitations. One disadvantage of the index FORT is related to its operator-dependent characteristic. It is unlikely to rule out the variation in marking AFC and PFC by different sonographers. Secondly, the association between FORT and the incidence of OHSS was not investigated in our study. Other limitations of our study lie in the retrospective and monocentric character, as well as the small study population. Prospective and multicentric investigations with larger simple size and longer duration of observation would be necessary to further validate the findings.

In summary, the present findings indicate that cumulative IVF outcomes have a positive correlation with FORT in PCOS patients when the FORT was less than 70%. For PCOS patients with polycystic ovarian morphology, ovulation disorder or hyperandrogenic manifestations, a high FORT could be conductive to achieving better pregnancy outcomes.

## Data availability statement

The raw data supporting the conclusions of this article will be made available by the authors, without undue reservation.

## Ethics statement

The studies involving human participants were reviewed and approved by the research ethics committee of the Second Hospital of Hebei Medical University. Written informed consent for participation was not required for this study in accordance with the national legislation and the institutional requirements.

## Author contributions

Study design: ZZ. Data acquisition: RJ, PC, and YL. Data analysis: RLJ, MC, and HH. Writing manuscript: Rulan Jiang. All authors read the article and agreed to the submitted version.
