# Supplementary material for: Effects of follicular output rate on cumulative clinical pregnancy rate and cumulative live birth rate in PCOS patients with different characteristics
Source: Front Endocrinol (Lausanne). 2022 Dec 19;13:1079502. doi: 10.3389/fendo.2022.1079502 (PMC9806261; doi:10.3389/fendo.2022.1079502)
Supplement: Supplementary file 1 [file Table_1.docx]

Supplementary Table 1. Univariate logistic regression analyse of cumulative clinical pregnancy and cumulative live birth

|  | Statistics | The cumulative pregnancy rate | | The cumulative live birth rate | |
| --- | --- | --- | --- | --- | --- |
|  |  | OR(95%CI) | *p*-value | OR(95%CI) | *p*-value |
| Age (years) | 28.555 ± 3.194 | 0.958 (0.877, 1.047) | 0.34643 | 0.955 (0.893, 1.020) | 0.16809 |
| BMI (kg/m^2^) | 25.250 ± 3.467 | 0.940 (0.866, 1.020) | 0.13794 | 0.995 (0.936, 1.058) | 0.86845 |
| Years of infertility (years) | 3.740 ± 2.390 | 0.976 (0.868, 1.096) | 0.67840 | 0.910 (0.835, 0.990) | 0.02903 |
| bFSH (mIU/mL） | 6.638 ± 1.791 | 0.917 (0.785, 1.071) | 0.27356 | 0.977 (0.868, 1.100) | 0.70174 |
| bE2 (pg/mL) | 49.166 ± 35.656 | 0.998 (0.991, 1.005) | 0.58557 | 1.002 (0.996, 1.008) | 0.55716 |
| bP (ng/mL) | 0.807 ± 0.604 | 0.735 (0.487, 1.108) | 0.14180 | 0.887 (0.632, 1.245) | 0.48804 |
| bLH (mIU/mL） | 9.270 ± 5.953 | 0.959 (0.917, 1.002) | 0.06263 | 0.971 (0.938, 1.005) | 0.09049 |
| bT (ng/mL) | 0.736 ± 0.493 | 0.842 (0.523, 1.355) | 0.47873 | 0.838 (0.567, 1.239) | 0.37513 |
| AMH (ng/mL) | 7.684 ± 4.384 | 0.977 (0.918, 1.040) | 0.46210 | 0.971 (0.927, 1.018) | 0.22150 |
| No. of AFC | 22.518 ± 4.261 | 1.022 (0.956, 1.091) | 0.52637 | 0.959 (0.911, 1.010) | 0.11161 |
| Starting dose of Gn (IU) | 175.633 ± 49.667 | 1.001 (0.995, 1.007) | 0.80556 | 1.002 (0.998, 1.006) | 0.35755 |
| Dose of Gn (IU) | 2288.887±1001.641 | 1.000 (1.000, 1.000) | 0.19460 | 1.000 (1.000, 1.000) | 0.57061 |
| Duration of Gn (IU) | 11.337 ± 3.285 | 0.964 (0.895, 1.038) | 0.33031 | 0.983 (0.924, 1.046) | 0.58584 |
| No. of PFC | 14.434 ± 5.862 | 1.065 (0.998, 1.136) | 0.05611 | 1.019 (0.980, 1.059) | 0.35237 |
| FORT | 65.687 ± 27.452 | 1.011 (0.998, 1.024) | 0.10493 | 1.007 (0.998, 1.016) | 0.10747 |
| Type of infertility |  |  |  |  |  |
| Primary | 287 (63.216%) | 1 |  | 1 |  |
| Secondary | 167 (36.784%) | 1.150 (0.629, 2.102) | 0.65051 | 1.288 (0.822, 2.018) | 0.26877 |
| Type of PCOS |  |  |  |  |  |
| A | 142 (31.278%) | 1 |  | 1 |  |
| B | 50 (11.013%) | 0.762 (0.309, 1.881) | 0.55555 | 0.873 (0.423, 1.801) | 0.71381 |
| C | 34 (7.489%) | 0.677 (0.247, 1.862) | 0.45017 | 0.623 (0.280, 1.384) | 0.24489 |
| D | 228 (50.220%) | 1.431 (0.734, 2.790) | 0.29296 | 1.149 (0.705, 1.874) | 0.57619 |
| PCOM |  |  |  |  |  |
| No | 50 (11.013%) | 1 |  | 1 |  |
| Yes | 404 (88.987%) | 1.520 (0.671, 3.440) | 0.31555 | 1.182 (0.613, 2.281) | 0.61772 |
| OAD |  |  |  |  |  |
| No | 34 (7.489%) | 1 |  | 1 |  |
| Yes | 420 (92.511%) | 1.701 (0.669, 4.321) | 0.26438 | 1.701 (0.813, 3.557) | 0.15846 |
| HA |  |  |  |  |  |
| No | 227 (50.000%) | 1 |  | 1 |  |
| Yes | 227 (50.000%) | 0.621 (0.346, 1.114) | 0.11022 | 0.791 (0.517, 1.210) | 0.27961 |
| Treatment plan |  |  |  |  |  |
| GnRH-a long protocol | 104 (22.907%) | 1 |  | 1 |  |
| GnRH-a prolonged protocol | 263 (57.930%) | 0.824 (0.400, 1.700) | 0.60089 | 0.671 (0.387, 1.164) | 0.15548 |
| GnRH-ant protocol | 87 (19.163%) | 1.025 (0.404, 2.600) | 0.95839 | 0.795 (0.401, 1.579) | 0.51248 |
| Number of transferred cycles |  |  |  |  |  |
| 1 | 416 (91.630%) | 1 |  | 1 |  |
| 2 | 38 (8.370%) | 0.861 (0.321, 2.311) | 0.76621 | 0.808 (0.387, 1.685) | 0.56938 |

Values are presented as number (%) or mean (SD).

BMI, body mass index; bFSH, basal follicle-stimulating hormone; bE2, baseline estradiol; bP, baseline progesterone; bT, baseline testosterone; bLH, baseline luteinizing hormone; AMH, anti-Mullerian hormone; Gn, Gonadotropin; PCOM, polycystic ovarian morphology; OAD, oligoanovulatory ovarian dysfunction; HA, hyperandrogenism.

Supplementary Table 2. The FORT values in different treatment plans

| Treatment plan | GnRH-a long protocol | GnRH-ant protocol | GnRH-a prolonged protocol | *p*-value |
| --- | --- | --- | --- | --- |
| N | 104 | 87 | 263 |  |
| FORT | 58.3300 (50.0000-70.8300) | 58.3300 (46.4450-77.0950) | 59.0900 (50.0000-75.0000) | 0.541 |

Continuous variables are presented as mean (SD) or median (interquartile range).
